# Improving behavioural compliance with the COVID-19 precautionary measures by means of innovative communication strategies: Social experimental studies

**DOI:** 10.1371/journal.pone.0272001

**Published:** 2022-07-28

**Authors:** Pepijn van Empelen, Katharina Preuhs, Leonhard A. Bakker, Petra Buursma, Rosa Andree, Charlotte Anraad, Hilde M. van Keulen

**Affiliations:** 1 TNO, Department of Child Health, Leiden, The Netherlands; 2 Department of Work & Social Psychology, Faculty of Psychology and Neuroscience, Maastricht University Maastricht, the Netherlands; Osaka University, JAPAN

## Abstract

**Objectives:**

Behavioural measures such as social distancing are crucial to prevent the spread of COVID-19. People sometimes have difficulty to comply with these behavioural measures for various reasons. Insight is needed into evidence-based strategies that can promote compliance. In the present study, we examined whether behaviour change techniques (volitional help sheet (VHS), behavioural journalism (BJ) and empathy inductions (EI)) could promote behavioural compliance with the COVID-19 measures.

**Methods:**

Three online experiments were carried out (N = 424–593) among Dutch adult citizens, using a randomized 2-group post-test and 1-week follow-up design. In each experiment, a control group was compared with the experimental condition (respectively VHS, BJ or EI condition).

**Results:**

Two out of the three different strategies did result in favourable changes with regard to the compliance-related measures. The VHS contributed to changes in perceived susceptibility of others (t = -2,78; f**2 = 0,019), perceived severity (t = -3,65; f**2 = 0,032) and individual behavioural compliance measures. People exposed to the VHS were more likely to receive less visitors (w = 16638; p = 0.003)and avoid crowds (w = 16631; p = 0.003). EI increased the perceived vulnerability of others. Video-based role model stories, based on BJ did not result in any changes.

**Conclusions:**

Behaviour change strategies may contribute to promoting behavioural compliance and could be used in public health communication. The empathy induction can be used to enhance other protection motives, while the volitional help sheet effectively can help people to overcome compliance barriers. Behavioural science can add to fighting the COVID-19 pandemic.

## Introduction

In many countries, governments have promoted behavioural measures, such as social distancing, avoiding crowded places, and hand hygiene measures to prevent the spread of COVID-19 and to protect vulnerable groups [1]. The effectiveness of these precautionary measures crucially depends on the compliance of the population. In the countries that imposed a lockdown, the rising number of hospital admissions and deaths has stagnated due to compliance with the precautionary measures [1]. However, as time passes people are less willing to comply with these measures [2]. As shown in previous virus outbreaks, perceived invulnerability, lack of motivation, and difficult situations or the perceived inability to carry out the behavioural measures can contribute to reduced compliance [3, 4]. Additionally, the amount of people going out in public areas was increasing due to the relaxation of the measures in some countries, the effects of which was shown in a second wave of infections [5]. In the Netherlands, the first COVID-19 patient was detected end of February 2020. An ‘intelligent’ lockdown was instigated as of March 12^th^ to slow down the infection rate. This lockdown implicated confinements to limit gatherings including more than 3 people until June 1^st^ 2020. Moreover, the population was urged to stay at home as much as possible, ‘non-vital’ shops had to close while others had to make sure to implement a social distancing policy with heavy fines for nonconformity [6]. Besides, restaurants, schools and children day-care had to close [7]. At the time of the study, in the Netherlands, the number of COVID-19 patients per day peaked in April 10th, and the number of deaths on March 31st.

A monitoring study among 50.291 Dutch citizens [8] pointed out that 91.4% of people would mind infecting someone with the virus. Furthermore, this study showed that most people support the measures, although the percentage of people who perceived the measures as effective has decreased between April 17^th^ and June 17^th^. For social distancing, the decrease in perceived effectiveness was the largest: approximately 12% in these three months. This is in line with the increase of people having difficulties to comply with certain measures, such as washing hands regularly and keeping a distance of 1.5 meters. In addition, fewer people indicated to be willing to follow these measures if they persist for several months. The number of people who are inclined to maintain the social distancing measure until December 2020 decreased by 19.6%. During the first wave of COVID-19 people felt generally vulnerable, yet experienced difficulties to comply with some behavioral measures, amongst others because of not having anticipated on difficult situations.

Behavioural change strategies can be used to motivate and support people to protect themselves and/or vulnerable others, with the aim to promote long-term compliance with the behavioural measures, in the absence of a vaccine. Behaviour change strategies have shown to contribute to a range of lifestyle changes and adherence behaviour [e.g., 9, 10]. There is, however, still no evidence to support the application of specific strategies to improve compliance with the COVID-19 measures. Behavioural models, such as the COM-B model, suggest that behavioural adherence is the result of capability, opportunity and motivation to enact a behaviour. Protection motivation, according to theories is the result of perceiving oneself or others being at risk of an undesirable health outcome, perceiving the benefits of precautionary measures and feeling capable to carry out these measures. Previously, such models have shown to be able to predict precautionary measures against infectious diseases [e.g., 4, 11]. Capability or self-efficacy refers to once ability to carry out a behaviour. Finally, opportunity refers to environment that facilitates the desired behaviour. Behavioural change strategies to increase motivational factors, capability and opportunities are likely to contribute to improved compliance with the behavioural measures. There are considerable number of behavior change strategies. In the present study we selected behavior change strategies that targeted the most important determinants of behavior and could be used practically (following the APEASE criteria (acceptability, practicability, effectiveness, affordability, spill-over effects and equity) [12].

## Present study

In the present study, we examined whether we could stimulate behavioural compliance with COVID-19 measures by implementing specific behaviour change strategies that can be used to target motivation, capabilities and opportunities, the key drivers of behaviour [13]. We carried out three experiments during the first lockdown between May and June 2020 in the Netherlands. In three experiments we tested three different behaviour change strategies. In the first experiment we examined whether a volitional help sheet could promote self-efficacy and behavioural compliance. Volitional help sheets, which are based on implementation intentions [14] have been shown to be effective in various health domains [15, 16]. They provide information on potentially difficult or challenging situations and state solutions for these situations (so-called IF-THEN planning). Thus, a volitional help sheet will help people to react in these specific situations and promote an automated response, in other words, to increase capability and opportunity for behaviour.

In the second experiment we examined whether behavioural journalism [17, 18] could improve motivation, self-efficacy (as a measure of capability) and behavioural compliance. Behavioural journalism is based on the theoretical idea of behavioural modelling [19] by means of authentic interviews with credible role models. Modelling is persuasive strategy in which people can learn from the behaviour and opinions of relevant others. The key idea is that by relating to the role models, who express their reason for protecting themselves from COVID-19, highlighting their means to do so, and showing positive outcomes, people would be able to identify with them. This could increase their motivation and feelings of ability to do so.

Lastly, we aspired to examine a behavioural method to increase prosocial motives to protect others. We aimed to examine whether empathy induction could promote salience of the need to protect vulnerable others, and increase the perceived responsibility to protect others [20]. Social psychologists have shown that helping behaviour is more likely when people: notice that something is going on, interpret the situation as being an emergency, feel a degree of responsibility, and implement the action choice. Particularly, the degree of responsibility can be enhanced by enhancing the feeling that a person is deserving of help [21]. In addition, studies on the promotion of helping behaviour have shown that promoting empathy/sympathy for a people in need of help can boost helping behaviour, but only when it is clear how helping behaviour can be realized [22]. Another aspect to promote social behaviour, is that people are motivated by their desire to get credit for their helping behaviour [23].

Inducing empathy and the felt need to help vulnerable groups (helper effect) may increase the motivation of people to comply with the measures. Studies on the promotion of helping behaviour have shown that promoting empathy for people in need of help can boost helping behaviour [22].

## Methods

Here we will provide information on the methods used for each of the different experiments (respectively study 1, 2 and 3). All experiments were approved by the TNO Institutional Review Board (2020–041).

### Study 1: Volitional help sheet

#### Methods and participants

In the present study it was hypothesized that the volitional help sheet would increase both self-efficacy and behavioural compliance with respect to the COVID-19 precautionary measures. The first study was carried out between May 10^th^ and May 23^rd^ 2020. During this period, the RIVM (National Institute for Public Health and the Environment) recommended complying with the following preventive measures: 1) washing one’s hands regularly (20 sec.) with water and soap, 2) sneezing in one’s elbow, 3) keeping 1.5m distance (except for people of the same household), 4) avoiding contact with vulnerable individuals (including people 70 years and older and/or with poor health), 5) staying at home as much as possible, 6) receiving as less visitors as possible, and 7) using paper towels. During data collection, preventive measures were supplemented with the advice to work from home as much as possible and avoid crowds. Incidences in the period of execution of the study ranged from 245 (10^th^ of May) to 176 (May 23^rd^) new daily infections.

Eligible participants were adults aged 18 years or older who lived in the Netherlands and mastered the Dutch language. Participants were recruited via a Facebook advertisement stating that the survey would take approximately 10 to 15 minutes to complete and that participants could win a €25 voucher. The advertisement was tailored to reach individuals living in the Netherlands with an age span ranging from 18 to 65 years.

We used a randomized post-test only design, with two follow-up measures (directly after the intervention and at 1-week follow-up). Participants who provided informed consent were randomized to the control or experimental group by random number allocation. Participants in both groups were asked to complete a survey measuring sociodemographic variables. Subsequently, the experimental group completed the volitional help sheet. Both groups ended their session with a post-test survey measuring self-efficacy and intention to comply with the precautionary measures, perceived COVID-19 susceptibility, perceived severity, perceived vulnerability of others, subjective behavioural compliance, and response efficacy. At 1-week follow-up, participants received a survey link by e-mail assessing self-efficacy to comply with the precautionary measures and behavioural compliance. Among those who completed the study, € 25,- vouchers were raffled.

#### Experimental manipulation

Participants allocated to the experimental group were provided with a volitional help sheet, including “if-then” statements, which comprised potential difficult situations to comply with the COVID-19 precautionary measures and corresponding possible solutions. The volitional help sheet presented 13 potential difficult situations and 20 different solutions (2–5 solutions per situation, with some solutions applying to various situations). These were previously gathered among the population via an online survey (n = 60).

These situations and solutions represented five COVID-19 preventive measures (e.g., washing your hands regularly (20 sec.) with water and soap). Difficult situations were for example, “If I can’t keep 1.5m distance while grocery shopping” or “If I do not have soap at home”, and solutions were e.g., “then I will move to a spot where I can keep enough distance” or “then I will put ’soap’ on my grocery shopping list”. Participants were asked to choose maximal three situations and one solution per situation. Participants then received a summary of their if-then plans. The volitional help sheet can be found in the supplementary material (see S1 Appendix). In total, 50 IF-THEN solutions were offered.

#### Measures

*Control variables*. In order to examine differences between the participants in the control and experimental conditions we gathered information on demographic background: age, gender, country of birth, highest completed level of education and whether they worked in the health care sector. In addition, we examined *self-reported COVID-19 infection* of participants with one question with 5 response categories (“Are you, or have you been infected with the coronavirus?”, with answers ranging from “I don’t know”, “Yes, I was tested”, “I think so, but I was not tested”, “No, I don’t think so” to “No, I was negatively tested”). Furthermore, participants were asked whether they had been in contact with someone who was infected with the virus, to be answered with “Yes”, “No” or “I don’t know”. Finally we assessed *Participation seriousness* was assessed at post-test and follow-up with one item on a 5-point Likert scale (“How seriously did you fill out the survey?”, 1 = not at all, to 5 = totally).

### Post-test and follow-up measures

Post-test measures included self-efficacy and intention to comply with COVID-19 precautionary measures, perceived susceptibility to get infected with COVID-19, perceived severity to get infected with COVID-19, perceived susceptibility of others to get infected with COVID-19, subjective behavioural compliance with COVID-19 measures and response efficacy to reduce the risk of infecting oneself and others. Items relating to self-efficacy, intention and behavioural compliance were based on the behavioral recommendations of the government. Constructs were assessed using a 5-point Likert scale. For each construct consisting of two or more items, the mean score was used in the regression analyses.

At 1 week follow-up, both self-efficacy to comply and behavioural compliance were assessed.

Items regarding self-efficacy and behavioural compliance were revised at follow-up due to changes in preventive measures recommended by the government (see Table 4).

*Self-efficacy* was assessed at post-test and follow-up with one item for each precautionary measure (e.g., “Do you feel able to wash your hands regularly (for 20 seconds)”; 1 = certainly not, to 5 = most certainly). The construct consisted of 7 items.

*Behavioural compliance* with the precautionary measures was assessed at follow-up with one item per precautionary measure (e.g., “In the last week I have sneezed or coughed in my elbow”, “In the last week I have avoided crowds”; 1 = never, to 5 = always). In total, behavioural compliance was assessed with 7 different items.

*Intention* to comply with the COVID-19 measures was determined at post-test with one item per precautionary measure (e.g., “In the next week, do you intend to keep 1.5 m away from other people?”; 1 = certainly not, to 5 = most certainly). The construct consisted of 7 items.

*Perceived susceptibility* to get infected with COVID-19 was assessed at post-test with two items (i.e., “What is the chance that you will become infected with the coronavirus the coming months?”, and “What is the chance you will get ill from the coronavirus the coming months?”; 1 = very small to 5 = very large).

*Perceived severity* was measured at post-test with 1 item (i.e., “How serious do you think it is to get infected with the coronavirus?”; 1 = not serious to 5 = very serious).

*Perceived susceptibility of others* towards getting infected with COVID-19 was measured at post-test with one item on a 5-point Likert scale (e.g., “How susceptible are [family members/ friends/ colleagues/ chronically ill/ neighbors] for the coronavirus?”, 1 = not at all susceptible, to 5 = very susceptible).

*Subjective behavioural compliance* was measured at post-test with one item on a 5-point Likert scale (“Do you comply with the precautionary measures in order to limit further spread of the coronavirus?”, 1 = certainly not, to 5 = most certainly).

*Response efficacy* was assessed at post-test with two items on a 5-point Likert scale (“The precautionary measures effectively reduce the risk of getting infected with the coronavirus” and “The precautionary measures reduce the risk of infecting others with the coronavirus”, 1 = certainly not, to 5 = most certainly).

#### A priori power analysis

It was calculated that in order to detect a small-to-medium effect size (Cohen’s d = 0.40 [10]; power of 0.8 and significance level of 0.05 (alpha)), and to account for 20% dropout, 125 participants per intervention arm per experiment were needed.

#### Statistical analyses

Differences in characteristics between the two groups were assessed using independent samples *t*-tests for continuous variables, Pearson’s chi-squared tests with Yates’ continuity correction for categorical variables, and Wilcoxon rank sum tests for ordered categorical variables. The differences in all psychological and behavioural constructs between the two groups were assessed using multiple linear regression models. The independent variables in these models were group, sex, age, and level of education. Effect sizes for linear regression models were calculated using Cohen’s *f*^*2*^statistic [24], $\left(R_{AB}^{2}-R_{A}^{2})/(1-R_{AB}^{2}\right)$, in which B is the variable of interest, i.e. the group variable, A is the set of all other variables, i.e., sex, age, and level of education, $R_{AB}^{2}$ is the proportion of variance accounted for by A and B together, and $R_{A}^{2}$ is the proportion of variance accounted for by A. To assess the differences between the two groups on the separate precautionary measures, Wilcoxon rank sum tests with continuity correction were used. The internal consistency of psychological and behavioural constructs was assessed using Cronbach’s coefficient alpha [25]. Spearman’s rank correlations were calculated, when the psychological construct was only measured with two items (i.e., *Perceived COVID-19 susceptibility*). All analyses were performed using R (version 3.6.1) [26]. For all regression analyses the distribution of the residuals was checked with a QQ plot. The residuals were normally distributed. To counter effects of multi comparisons problem, we used Holm-Bonferroni correction.

### Study 2: Behavioural journalism

In experiment two, again, participants were recruited via social media (similar to study 1) and randomly allocated to the experimental or control condition. Participants in the experimental group were offered the behavioural journalism manipulation based on four short films, in which “positive role models” explained how they dealt with the covid compliance measures during times of COVID-19. It was hypothesized that behavioral journalism strategy would increase feelings of capability (as measured by self-efficacy) and motivation (as measured by intention).

#### Method and participants

We again used a randomized post-test only design with two post-tests (directly after the intervention and at 1-week follow-up). The second study took place between 15^th^ of May and 7^th^ of June 2020. Preventive measures at the time of execution comprised washing one’s hands regularly (20 sec.) with water and soap, sneezing in one’s elbow, keeping 1.5m distance (except for people of the same household), avoiding contact with vulnerable individuals (including people 70 years and older and/or with poor health), using paper towels, working from home as much as possible and avoiding crowds. The respective incidence during the time the study was executed ranged between 200 (15^th^ of May) and 239 (7^th^ of June 2020).

#### Experimental manipulation

Participants in the behavioural journalism condition were offered four short films, ranging from 1:22 minutes to 1:40 minutes. The four films comprised: a male student, a young working couple, a pregnant woman and a healthcare worker. In all scenarios the depicted individuals shared the impact that COVID-19 has on their lives and how they dealt with it. This included taking precautionary measures when doing everyday errands and during daily activities. Furthermore, the role models explained why they believed it to be important to comply with the precautionary measures. Participants were instructed to watch at least one of the films while being allowed to watch as many of the role model stories as they felt seemed relevant or interesting to them.

**Fig 1:**
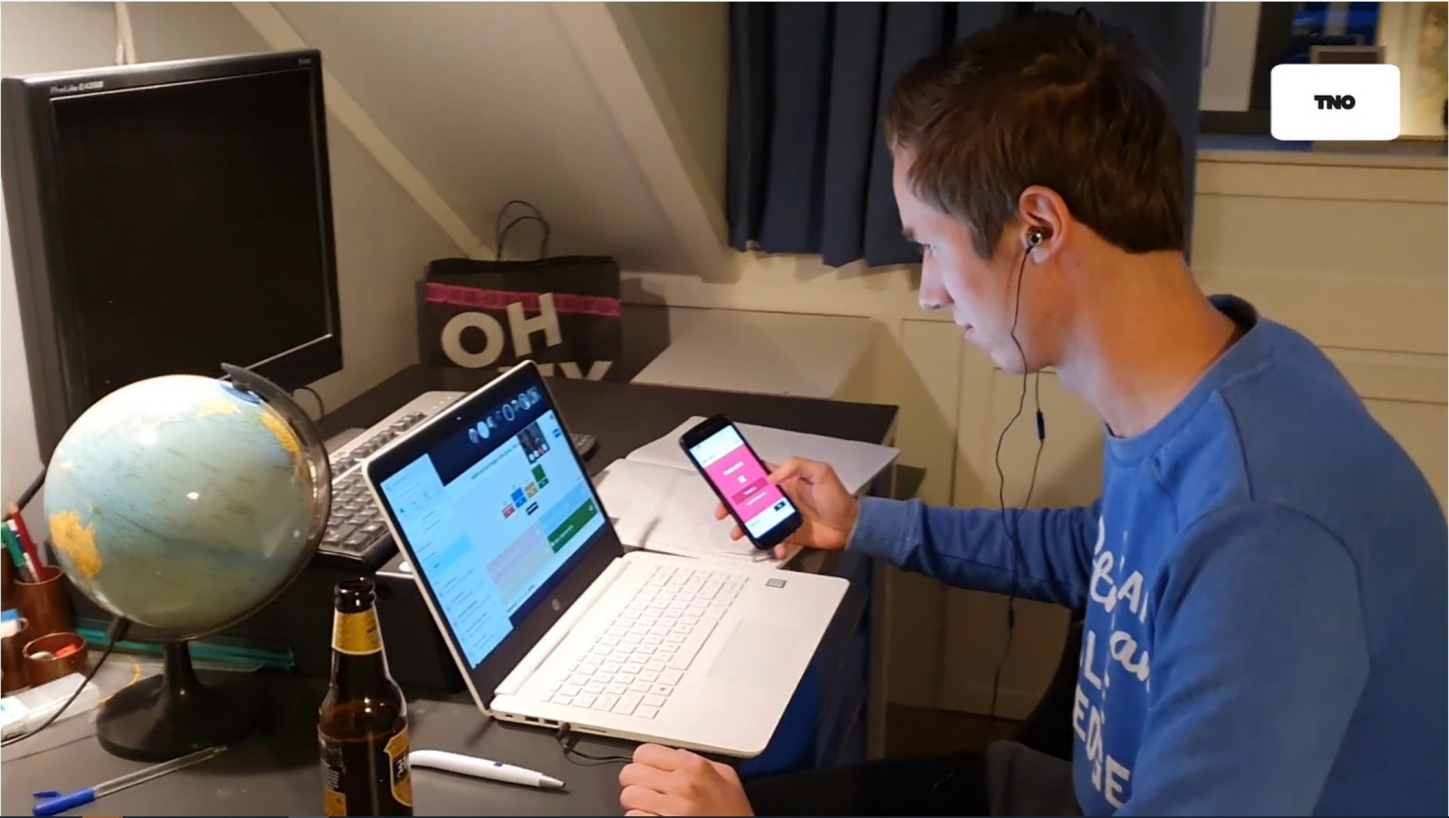
Screenshot scenario 1. Depicting student Maarten, 21 years, “meeting his friends online”.

*Scenario 1 (1*:*25 minutes)*. In the first scenario, a young male student (21 years) reports how he accommodates to his new lifestyle in times of COVID-19: Meeting his friends online for pub quizzes and attending online lectures. Furthermore, the student tells that he is currently unable to visit his grandparents. Yet, he states his problems not to be as big as he ‘only’ needs to stay at home whereas others are suffering far more from the impact COVID-19 has on their lives, highlighting the need to comply with the precautionary measures. Besides emphasizing the importance of staying healthy, the young man ends on a positive note, elaborating that the current situation can be seen as a chance to do and learn new things from home (Fig 1). (The individual in this manuscript has given written informed consent (as outlined in PLOS consent form) to publish these case details).

*Scenario 2 (1*:*40 minutes)*. The second scenario depicts a young couple who reveal what their everyday life looks like under the current circumstances: While adapting their lives in accordance with the precautionary measures and missing out on social contacts seemed to be hard at first, the couple reports changes to their daily rhythm including running errands alone, working from home and eventually being in touch/connected with others even more than before. They explain that they comply with the regulations to prevent infecting vulnerable others such as their (grand)parents, thereby advising on being creative to find something that is energizing and one enjoys like trying new recipes.

*Scenario 3 (1*:*35 minutes)*. The third scenario is about a pregnant woman. The young mother talks about how she and her partner now work from home and take care of their son together. Apart from doing pregnancy yoga via zoom in her living room, she talks about getting to know their neighbourhood in a different way through taking a lot of walks. The role model reports that she and her family comply with the regulations to not infect vulnerable others. Also, she reports that working from home provides her with peace of mind and that her son’s development enormously benefitted from the prolonged time at home as the toddler now talks more than before and immensely enjoyed the time spent with his parents.

*Scenario 4 (1*:*22 minutes)*. In the last scenario, the viewer accompanies a young woman working as specialist in geriatric medicine to her workplace, where she helps vulnerable elderly suffering from physical and mental health problems such as dementia. Whilst one of the greatest changes in her daily work life is the lack of visitors her patients would normally receive, she talks about seeing her patients less often and spending time in the office more frequently than she normally would. Before working with infected patients, she has to get changed and is very alert not to infect any other patient, which is one of her fears. Simultaneously, to persevere, the health care worker reports to remind herself that she is complying with the regulations for her patients.

#### Measures

Similar demographic variables as in the first experiment were included as part of a baseline questionnaire. Items regarding self-efficacy, intention to comply with precautionary measures and behavioural compliance thereof were adapted to the most current behavioural measures due to governmental revisions of precautionary measures. Behavioural compliance was therefore assessed with 7 items. All other constructs were identical to study 1 (see Table 2).

*Manipulation check*. In addition, the films were evaluated in the experimental group, by asking participants at post-test how many of the films they watched, and whether they perceived the role model stories as relevant, recognizable and helpful (1 = not at all, to 5 = very relevant/recognizable/helpful).

### Post-test and follow-up measures

Measures were similar to those of study 1, except that the items regarding self-efficacy, intention to comply with precautionary measures and behavioural compliance were adapted to comply to the most current behavioural measures due to governmental revisions of precautionary measures. Behavioural compliance was therefore assessed with 7 items. Like study 1 we assessed all measures at post-test, and self-efficacy, intention and behaviours at 1-week follow-up.

### Study 3: Empathy induction

#### Method and participants

In this experiment we assessed whether empathy induction could promote salience of the need to protect vulnerable others and increase the perceived responsibility to protect others. We used a randomized post-test only design with two post-test measures (directly and at 1-week follow-up). In this experiment, participants were randomized to the experimental condition, which received the empathy induction or the control group.

The third study took place between 16^th^ of May and 7^th^ of June 2020. Preventive measures at the time of execution comprised washing one’s hands regularly (20 sec.) with water and soap, sneezing in one’s elbow, keeping 1.5m distance (except for people of the same household), avoiding contact with vulnerable individuals (including people 70 years and older and/or with poor health), using paper towels, working from home as much as possible and avoiding crowds. The respective incidence during the time the study was executed ranged between 189 (16^th^ of May) and 239 (7^th^ of June 2020).

The recruitment of participants and procedure were identical to those of study 1, except that the advertisement was set to reach individuals ranging from 18 to 40 years. This change was made in order to reach members of the younger generation, who were expected to protect the more vulnerable members of society, including elderly citizens.

#### Experiment: Empathy induction

**Fig 2:**
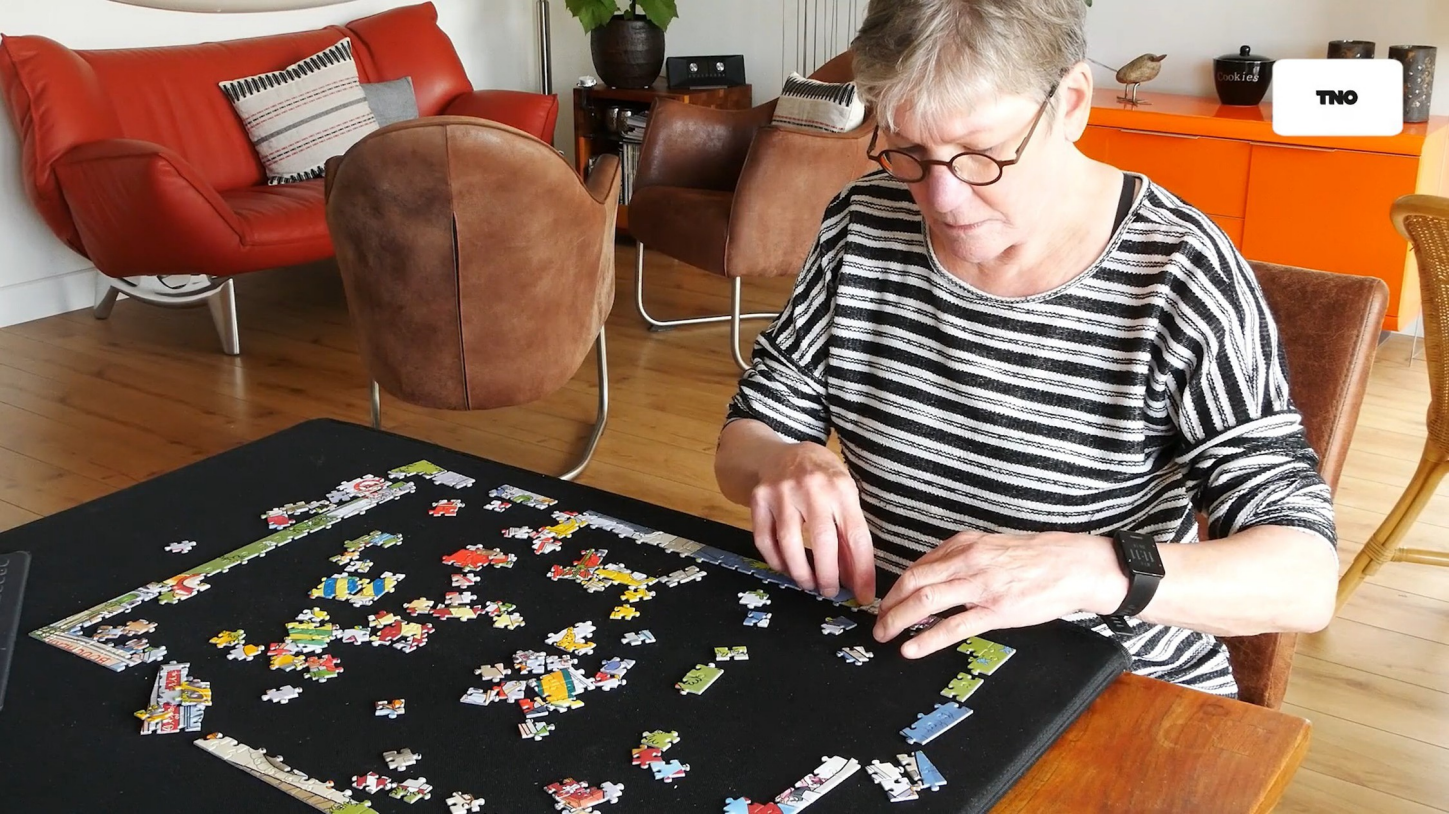
Screenshot short film. Showing Anne-Marie, a 70-year old woman during activities in times of corona.

Participants allocated to the experimental group were asked to watch a short film (1:42 min.) depicting a 70-year old woman who explains why she belongs to the at-risk population, due to her age and having asthma (Fig 2) (The individual in this manuscript has given written informed consent (as outlined in PLOS consent form) to publish these case details). In the video, the woman points out that, while she is following the precautionary measures herself to keep safe from COVID-19, she is still dependent on others to follow the measures to be protected. Participants who indicated their readiness to protect others were offered a gift as credit for wanting to do so. The gift also served as a reminder for taking precautionary measures and comprised a blue silicone band stating “Door mij coronavrij!” [Corona-free through me!”].

#### Measures

Constructs were the similar to those used in Study 2 (see Table 2). In addition, empathy induction and attitude to protect others were measured using a 5-point Likert scale.

*Empathy induction* was assessed at post-test after the intervention with five items (“Which feelings did watching the short film with Anne-Marie evoke?” with respect to “Sympathy”, “Compassion”, “Involvement”, “Empathy” and “Warmth”, 1 = not at all, to 5 = absolutely).

*Attitude towards protecting others* was assessed at post-test with three items (“We should protect vulnerable people from corona”, 1 = totally disagree, to 5 = totally agree, “How important do you think it is to prevent vulnerable people to get infected with corona?”, 1 = totally unimportant, to 5 = totally important, and “To what extent are you willing to protect another by following the precautionary measures?”, 1 = not at all, to 5 = totally). Individuals reporting a certain extent to which they are willing to protect another were offered to receive a small gift to get credit for their behaviour.

*Manipulation check*. Participants in the experimental group were asked to indicate whether they had watched the film along with feelings evoked by watching it, as well as their attitude towards, and willingness to protect others. In addition, participants in the experimental group were asked to indicate whether they had requested, received and worn the gift.

## Results

### Study 1: Volitional help sheet

In total, 424 of 482 participants consented to participate, who were allocated to the intervention (n = 181) or control (n = 243) group. Data of 339 participants were analysed (n = 149 intervention, n = 190 control). Dropout was 21%, mainly due to non-response or unreachable e-mail addresses.

The characteristics of participants are presented in Table 1. Participants in the intervention and control group were similar in socio-demographical characteristics, with only a difference in level of education between the two groups. Most participants were female, were born in the Netherlands, did not work in healthcare and had someone in their environment with an increased risk of becoming ill from COVID-19.

**Table 1:** Characteristics of participants in the three experiments.

Variable		VHS experiment					BHJ experiment					SI experiment				
		Intervention group		Control group		*p*-value	Intervention group		Control group		*p-*value	Intervention group		Control group		*p*-value
		(*n* = 181)		(*n* = 243)			(*n* = 290)		(n = 302)			(n = 260)		(n = 316)		
Age, *mdn* (*iqr*)		39	(34–55)	38	(34–49)	0.38	38	(32–53)	38	(32–51)	0.82	38	(33–45)	37	(33–41)	0.04
Sex, *n* (%)						0.10*					0.04*					0.42*
	Female	154	(85.1)	190	(78.2)		218	(75.2)	250	(82.8)		177	(68.1)	227	(71.8)	
	Male	27	(14.9)	53	(21.8)		70	(24.1)	51	(16.9)		80	(30.8)	87	(27.5)	
	Other	0	(0.0)	0	(0.0)		0	(0.0)	1	(0.3)						
	I prefer not to say	0	(0.0)	0	(0.0)		2	(0.7)	0	(0.0)		3	(1.2)	2	(0.6)	
Level of education, *n* (%)						0.04					0.47					0.95
	Low	35	(19.4)	40	(16.5)		41	(14.2)	40	(13.3)		19	(7.3)	29	(9.3)	
	Intermediate	81	(45.0)	89	(36.6)		79	(27.3)	97	(32.2)		80	(30.9)	89	(28.4)	
	High	64	(35.6)	114	(46.9)		169	(58.5)	164	(54.5)		160	(61.8)	195	(62.3)	
	Unknown	1		0			1		1			1		3		
Country of birth, *n* (%*)*						0.90					0.74					0.68
	Netherlands	173	(95.6)	234	(96.3)		270	(93.8)	280	(92.7)		245	(94.2)	294	(93.0)	
	Other	8	(4.4)	9	(3.7)		18	(6.2)	22	(7.3)		15	(5.8)	22	(7.0)	
	Unknown	0		0			2		0			0		0		
One or more children living at home, n (%)						0.89					0.65					0.81
	Yes	69	(38.1)	95	(39.3)		108	(37.2)	119	(39.4)		116	(44.8)	136	(43.5)	
	No	112	(61.9)	147	(60.7)		182	(62.8)	183	(60.6)		143	(55.2)	177	(56.5)	
	Unknown	0		1			0		0			1		3		
Are you / have you been infected with the corona virus, *n* (%)						0.03					0.83					0.25
	I don’t know	38	(21.0)	67	(27.6)		74	(25.5)	72	(23.8)		52	(20.0)	87	(27.5)	
	Yes, I have been tested	1	(0.6)	2	(0.8)		3	(1.0)	1	(0.3)		4	(1.5)	6	(1.9)	
	I think so, but I have not been tested	25	(13.8)	53	(21.8)		48	(16.6)	51	(16.9)		56	(21.5)	60	(19.0)	
	I don’t think so	112	(61.9)	116	(47.7)		159	(54.8)	170	(56.3)		145	(55.8)	157	(49.7)	
	No, I have been tested	5	(2.8)	5	(2.1)		6	(2.1)	8	(2.6)		3	(1.2)	6	(1.9)	
Have you been in contact with someone who is or has been infected, *n* (%)						0.33					0.73					0.46
	Yes	31	(17.1)	56	(23.0)		63	(21.7)	65	(21.5)		57	(21.9)	83	(26.3)	
	No	67	(37.0)	82	(33.7)		106	(36.6)	102	(33.8)		97	(37.3)	108	(34.2)	
	I don’t know	83	(45.9)	105	(43.2)		121	(41.7)	135	(44.7)		106	(40.8)	125	(39.6)	
Has someone in his/her environment with an increased risk of becoming seriously ill from the corona virus, *n* (%)						0.39					0.87					1.00
	Yes	120	(66.3)	150	(61.7)		170	(58.6)	180	(59.6)		149	(57.3)	182	(57.6)	
	No	61	(33.7)	93	(38.3)		120	(41.4)	122	(40.4)		111	(42.7)	134	(42.4)	
Works in healthcare, *n* (%)						1.00					0.78					0.80
	Yes	42	(23.2)	57	(23.5)		55	(19.0)	61	(20.2)		39	(15.0)	51	(16.1)	
	No	139	(76.8)	186	(76.5)		235	(81.0)	241	(79.8)		221	(85.0)	265	(83.9)	
How seriously did you complete this questionnaire, *n* (%) †						0.35					0.09					0.75
	Not seriously at all	0	(0.0)	0	(0.0)		1	(0.3)	0	(0.0)		0	(0.0)	0	(0.0)	
	Somewhat seriously	0	(0.0)	0	(0.0)		0	(0.0)	1	(0.3)		1	(0.4)	0	(0.0)	
	Neutral	0	(0.0)	1	(0.5)		7	(2.4)	4	(1.3)		3	(1.2)	1	(0.3)	
	Somewhat seriously	12	(8.1)	20	(10.5)		27	(9.3)	19	(6.3)		14	(5.4)	19	(6.0)	
	Very seriously	137	(91.9)	169	(88.9)		255	(87.9)	278	(92.1)		242	(93.1)	296	(93.7)	
	Missing	32		53			0		0			0		0		

*Note*. *iqr* interquartile range, ISCED International standard classification of education (2011), *mdn* median, *n* number of observations.

Level of education was coded as Low = ISCED 0–4, Intermediate = ISCED 5–6, High = ISCED 7–8. % calculated based on non-missing data

* Pearson chi-squared test only included males and females.

† measured at T1

A manipulation check was performed for the VHS. Participants selected situations that they perceived to be challenging. In total, 58.0% of the participants perceived it as challenging ‘when there is not enough space to pass someone in the supermarket’. Most participants reported that they would either wait until there is enough space or to step aside to create enough space as solution. Furthermore 56.4% of the participants declared to experience it as challenging ‘when family members feel lonely and ask if I come by’. Most common solutions to this situation were to visit them, but only outside and with enough distance (60.8%) or to videocall those family members (32.4%). For the full list, see S1 Appendix.

Tables 2 and 3 present the means, standard deviations, and internal consistency of the constructs and the results of the regression analyses with the uncorrected p-values. The regression analyses (with condition as predictor, controlled for sex, age, and level of education) indicated that participants who received the volitional help sheet showed more favourable results on most measures. At post-test, participants in the intervention group had a higher self-efficacy and intention to comply with the precautionary measures compared to the control group. In addition, participants in the intervention group had a higher perceived compliance, a higher perceived susceptibility towards themselves and a higher perceived vulnerability of others to become infected with COVID-19. Lastly, participants in the intervention group reported a higher perceived severity of becoming infected with COVID-19.

**Table 2:** Descriptive statistics and Cronbach’s coefficient alpha for measures in the three experiments.

	Tn	Construct	Intervention group			Control Group			*α*	95% CI	
Experiment			*n*	*m*	*(sd)*	*n*	*m*	*(sd)*		(lower;	upper)
VHS	0	Self-efficacy	181	4.15	(0.71)	243	3.94	(0.79)	0.76	(0.72;	0.79)
		Intention	179	4.22	(0.79)	238	3.99	(0.93)	0.84	(0.81;	0.86)
		Perceived COVID-19 susceptibility †	181	2.73	(1.02)	243	2.49	(0.89)	0.76	(0.69;	0.82)
		Perceived severity §	181	3.80	(1.07)	243	3.33	(1.14)	-	-	-
		Perceived susceptibility (others)	181	3.61	(0.62)	243	3.39	(0.73)	0.79	(0.75;	0.82)
		Perceived compliance §	181	4.09	(0.76)	243	3.85	(1.03)	-	-	-
		Response efficacy †	181	3.92	(0.95)	243	3.76	(1.19)	0.82	(0.75;	0.86)
	1	Behaviour index	149	4.03	(0.72)	190	3.88	(0.80)	0.83	(0.81;	0.86)
		Self-efficacy	149	4.10	(0.77)	190	3.95	(0.84)	0.81	(0.79;	0.84)
BHJ	0	Self-efficacy	290	4.01	(0.77)	302	3.94	(0.73)	0.71	(0.67;	0.74)
		Intention	284	4.08	(0.86)	299	4.02	(0.87)	0.80	(0.78;	0.82)
		Perceived COVID-19 susceptibility †	290	2.44	(0.89)	302	2.48	(0.88)	0.75	(0.69;	0.80)
		Perceived severity §	290	3.45	(1.15)	302	3.46	(1.06)	-	-	-
		Perceived susceptibility (others)	290	3.42	(0.73)	302	3.44	(0.74)	0.82	(0.79;	0.84)
		Perceived compliance §	289	3.95	(1.04)	301	4.06	(0.93)	-	-	-
		Response efficacy †	290	3.91	(1.17)	302	3.84	(1.16)	0.88	(0.85;	0.91)
	1	Behaviour index	212	4.08	(0.71)	235	4.07	(0.65)	0.70	(0.66;	0.74)
		Self-efficacy	212	4.03	(0.79)	235	4.04	(0.68)	0.72	(0.68;	0.75)
SI	0	Self-efficacy	260	3.76	(0.93)	316	3.69	(0.92)	0.80	(0.78;	0.83)
		Intention	258	3.82	(1.05)	310	3.67	(1.07)	0.86	(0.84;	0.88)
		Perceived COVID-19 susceptibility †	260	2.33	(0.94)	316	2.27	(0.95)	0.75	(0.69;	0.80)
		Perceived severity §	260	3.18	(1.16)	316	3.03	(1.15)	-	-	-
		Perceived susceptibility (others)	260	3.32	(0.72)	316	3.18	(0.78)	0.81	(0.78;	0.83)
		Perceived compliance §	260	3.93	(1.13)	315	3.79	(1.16)	-	-	-
		Response efficacy †	260	3.39	(1.40)	316	3.44	(1.35)	0.85	(0.81;	0.88)
		Feelings	260	3.85	(0.92)	-	-	-	0.92	(0.92;	0.93)
		Statements	260	3.88	(1.07)	-	-	-	0.79	(0.76;	0.82)
	1	Behaviour index	196	3.85	(0.82)	232	3.79	(0.87)	0.81	(0.78;	0.83)
		Self-efficacy	196	3.84	(0.88)	232	3.79	(0.93)	0.82	(0.80;	0.84)

**Table 3:** Results of regression analyses for the three experiments.

Dependent variable	Tn	Independent variable	VHS experiment						BHJ experiment						SI experiment					
			*b*	*se*	*t*	*p*	*R* ^*2*^	*f* ^*2*^	*b*	*se*	*t*	*p*	*R* ^*2*^	*f* ^*2*^	*b*	*se*	*t*	*p*	*R* ^*2*^	*f* ^*2*^
Self-efficacy	T_0_	Intercept	4.22	0.17	25.47	<0.001	0.08	0.015	3.84	0.14	27.06	<0.001	0.07	0.005	3.85	0.19	19.95	<0.001	0.17	0.005
		Group	-0.18	0.07	-2.52	0.012			-0.10	0.06	-1.74	0.083			-0.12	0.07	-1.63	0.103		
		Sex. Male	-0.44	0.09	-4.75	<0.001			-0.41	0.07	-5.48	<0.001			-0.57	0.08	-7.31	<0.001		
		Age	0.00	0.00	1.09	0.279			0.01	0.00	2.32	0.021			-0.01	0.00	-2.36	0.019		
		Level of education. Intermediate	-0.17	0.10	-1.63	0.105			-0.06	0.10	-0.65	0.516			0.17	0.14	1.23	0.218		
		Level of education. High	-0.14	0.10	-1.35	0.177			0.13	0.92	1.43	0.155			0.59	0.13	4.49	<0.001		
Intention	T_0_	Intercept	4.28	0.19	22.09	<0.001	0.07	0.015	3.86	0.17	23.05	<0.001	0.06	0.003	3.99	0.22	17.82	<0.001	0.16	0.011
		Group	-0.21	0.09	-2.48	0.014			-0.09	0.07	-1.23	0.220			-0.20	0.08	-2.46	0.014		
		Sex. Male	-0.47	0.11	-4.34	<0.001			-0.39	0.09	-4.46	<0.001			-0.61	0.09	-6.67	<0.001		
		Age	0.00	0.00	0.91	0.360			0.01	0.00	2.54	0.011			-0.01	0.00	-2.87	0.004		
		Level of education. Intermediate	-0.15	0.12	-1.24	0.215			-0.12	0.12	-1.03	0.303			0.24	0.16	1.52	0.130		
		Level of education. High	-0.11	0.12	-0.90	0.368			0.12	0.11	1.09	0.277			0.66	0.15	4.37	<0.001		
Perceived COVID- 19 susceptibility	T_0_	Intercept	2.64	0.21	12.42	<0.001	0.04	0.013	2.11	0.17	12.43	<0.001	0.04	0.000	2.33	0.21	11.28	<0.001	0.09	0.002
		Group	-0.22	0.09	-2.37	0.018			0.00	0.07	-0.04	0.972			-0.08	0.08	-1.04	0.300		
		Sex. Male	-0.20	0.12	-1.72	0.085			-0.27	0.09	-3.10	0.002			-0.26	0.08	-3.15	0.002		
		Age	0.00	0.00	-0.63	0.532			0.00	0.00	0.54	0.589			-0.01	0.00	-2.96	0.003		
		Level of education. Intermediate	0.29	0.13	2.20	0.028			0.35	0.12	2.98	0.003			0.36	0.15	2.42	0.016		
		Level of education. High	0.23	0.13	1.69	0.091			0.42	0.11	3.81	<0.001			0.64	0.14	4.58	<0.001		
Perceived severity	T_0_	Intercept	3.33	0.24	13.75	<0.001	0.13	0.032	2.52	0.21	12.04	<0.001	0.08	0.000	3.28	0.25	12.91	<0.001	0.09	0.009
		Group	-0.39	0.11	-3.65	<0.001*			-0.03	0.09	-0.40	0.692			-0.21	0.09	-2.29	0.023		
		Sex. Male	-0.41	0.13	-3.07	0.002			-0.36	0.11	-3.34	<0.001			-0.63	0.10	-6.13	<0.001		
		Age	0.02	0.00	4.29	<0.001			0.02	0.00	6.46	<0.001			0.00	0.00	-1.05	0.293		
		Level of education. Intermediate	-0.18	0.15	-1.20	0.231			0.13	0.14	0.89	0.374			0.18	0.18	0.99	0.325		
		Level of education. High	-0.40	0.15	-2.61	0.009			0.17	0.14	1.22	0.221			0.40	0.17	2.32	0.021		
Perceived susceptibility (others)	T_0_	Intercept	3.32	0.15	21.73	<0.001	0.06	0.019	2.70	0.14	19.65	<0.001	0.09	0.000	3.64	0.16	22.34	<0.001	0.10	0.014
		Group	-0.19	0.07	-2.78	0.006*			-0.02	0.06	-0.32	0.751			-0.17	0.06	-2.85	0.005*		
		Sex. Male	-0.24	0.08	-2.80	0.005			-0.31	0.07	-4.36	<0.001			-0.38	0.07	-5.69	<0.001		
		Age	0.01	0.00	2.45	0.015			0.01	0.00	5.36	<0.001			-0.01	0.00	-2.17	0.030		
		Level of education. Intermediate	0.09	0.10	0.92	0.357			0.34	0.09	3.60	<0.001			-0.10	0.12	-0.90	0.370		
		Level of education. High	0.02	0.10	0.24	0.810			0.37	0.09	4.18	<0.001			0.13	0.11	1.19	0.234		
Perceived compliance	T_0_	Intercept	3.69	0.21	17.72	<0.001	0.03	0.014	3.43	0.19	17.88	<0.001	0.02	0.002	3.26	0.26	12.68	<0.001	0.06	0.005
		Group	-0.22	0.09	-2.45	0.015			0.09	0.08	1.12	0.263			-0.16	0.09	-1.71	0.088		
		Sex. Male	-0.10	0.12	-0.84	0.400			-0.12	0.10	-1.19	0.234			-0.21	0.10	-2.03	0.043		
		Age	0.01	0.00	1.73	0.084			0.01	0.00	3.03	0.003			0.00	0.00	0.35	0.731		
		Level of education. Intermediate	0.20	0.13	1.54	0.121			0.12	0.13	0.87	0.385			0.57	0.18	3.10	0.002		
		Level of education. High	0.15	0.13	1.15	0.251			0.23	0.12	1.87	0.063			0.83	0.17	4.81	<0.001		
Response efficacy	T_0_	Intercept	4.01	0.25	16.27	<0.001	0.02	0.003	3.20	0.22	14.36	<0.001	0.06	0.002	4.15	0.29	14.43	<0.001	0.16	0.000
		Group	-0.13	0.11	-1.22	0.223			-0.10	0.09	-1.09	0.278			-0.02	0.11	-0.20	0.842		
		Sex. Male	-0.35	0.14	-2.58	0.010			-0.32	0.12	-2.72	0.007			-0.68	0.12	-5.78	<0.001		
		Age	0.00	0.00	0.21	0.835			0.01	0.00	3.15	0.002			-0.02	0.00	-4.75	<0.001		
		Level of education. Intermediate	-0.09	0.15	-0.60	0.549			0.09	0.15	0.60	0.550			0.06	0.21	0.28	0.776		
		Level of education. High	-0.10	0.16	-0.64	0.521			0.54	0.14	3.77	<0.001			0.64	0.19	3.28	0.001		
Behaviour index	T_1_	Intercept	4.15	0.19	21.46	<0.001	0.07	0.005	3.87	0.16	24.70	<0.001	0.05	0.001	4.15	0.22	19.11	<0.001	0.13	0.003
		Group	-0.11	0.08	-1.29	0.198			-0.03	0.06	-0.50	0.619			-0.08	0.08	-1.10	0.274		
		Sex. Male	-0.44	0.10	-4.28	<0.001			-0.32	0.08	-4.04	<0.001			-0.45	0.09	-5.16	<0.001		
		Age	0.00	0.00	0.52	0.606			0.00	0.00	2.03	0.043			-0.01	0.00	-2.69	0.007		
		Level of education. Intermediate	-0.18	0.12	-1.47	0.143			0.01	0.11	0.09	0.932			0.00	0.16	-0.01	0.989		
		Level of education. High	-0.12	0.12	-0.99	0.320			0.12	0.10	1.20	0.230			0.34	0.16	2.16	0.031		
Self-efficacy	T_1_	Intercept	4.13	0.20	20.40	<0.001	0.1	0.003	3.85	0.17	22.62	<0.001	0.03	0.000	4.03	0.23	17.18	<0.001	0.11	0.002
		Group	-0.08	0.09	-0.95	0.342			-0.01	0.07	-0.10	0.921			-0.07	0.08	-0.87	0.388		
		Sex. Male	-0.57	0.11	-5.27	<0.001			-0.27	0.08	-3.20	0.001			-0.47	0.10	-4.98	<0.001		
		Age	0.00	0.00	1.50	0.135			0.00	0.00	1.85	0.065			-0.01	0.00	-1.73	0.084		
		Level of education. Intermediate	-0.21	0.13	-1.62	0.107			-0.04	0.12	-0.35	0.725			0.01	0.18	0.03	0.974		
		Level of education. High	-0.20	0.13	-1.51	0.131			0.09	0.11	0.83	0.405			0.33	0.17	1.94	0.053		

Note. *b* unstandardized regression coefficient, *f*^*2*^ Cohen’s *f*^*2*^ effect size, *p p*-value, *R*^*2*^ explained variance, *se* standard error, *t t*-value. *significant after Holm-Bonferroni correction).

When we adjusted for multiple testing by means of the Holm-Bonferroni correction, the differences in perceived severity and perceived susceptibility of others remained significant.

At follow-up, participants in the intervention group more often complied with several individual precautionary measures compared to the control group. Participants reported to comply more often to sneezing and coughing in the elbow, staying at home as much as possible, receiving as little visitors as possible and avoiding crowds compared to the control group (see Table 4). No significant differences were found between conditions on the behavioural compliance index and self-efficacy at follow-up. When adjusting for multiple comparison by means of the Holm-Bonferroni method, only the effects for receiving as little visitors ***as*** possible, and avoiding crowds remain.

**Table 4:** Descriptive statistics of the behavioural measures for the three experiments.

		Intervention group				Control group				Wilcoxon rank sum	
		*m*	*(sd)*	*mdn*	*(iqr)*	*m*	*(sd)*	*mdn*	*(iqr)*	*W*	*p*
**Preventive behaviour VHS**											
1.	Wash my hands regularly (20 sec.) with soap and water	4.11	(0.79)	4	(4–5)	3.78	(1.00)	4	(3–5)	15755	0.056
2.	Sneeze or cough in my elbow	4.37	(1.01)	5	(4–5)	4.25	(0.91)	4	(4–5)	15885	0.032
3.	Keep 1.5 meters away from other people	4.03	(0.80)	4	(4–5)	3.93	(0.91)	4	(4–4)	14883	0.366
4.	Avoid people who are vulnerable	4.23	(1.09)	5	(4–5)	4.12	(1.11)	4	(4–5)	14992	0.309
5.	Stay at home as much as possible	3.79	(1.11)	4	(3–4)	3.49	(1.22)	4	(3–4)	16238	0.014
6.	Receive as little visitors as possible	4.06	(1.05)	4	(4–5)	3.76	(1.09)	4	(3–4)	16638	0.003*
7.	Use paper tissues	4.34	(0.98)	5	(4–5)	3.98	(1.20)	4	(4–5)	14708	0.478
8.	Working from home as much as possible	3.16	(1.79)	4	(1–5)	3.42	(1.70)	4	(1–5)	13096	0.212
9.	Avoid crowds	4.34	(0.98)	5	(4–5)	3.98	(1.20)	4	(4–5)	16631	0.003*
**Preventive behaviour BHJ**											
1.	Wash my hands regularly (20 sec.) with soap and water	4.09	(0.93)	4	(4–5)	4.11	(0.91)	4	(4–5)	24747	0.898
2.	Sneeze or cough in my elbow	4.41	(0.91)	5	(4–5)	4.36	(0.94)	4	(4–5)	25568	0.584
3.	Keep 1.5 meters away from other people	4.01	(0.85)	4	(4–5)	4.02	(0.75)	4	(4–4)	25219	0.801
4.	Avoid people who are vulnerable	4.19	(1.02)	4	(4–5)	4.12	(1.02)	4	(4–5)	26087	0.352
5.	Work from home as much as possible	3.59	(1.72)	5	(1–5)	3.46	(1.70)	1	(1–5)	26406	0.239
6.	Avoid crowds	4.24	(0.92)	4	(4–5)	4.24	(0.91)	4	(4–5)	24869	0.974
7.	Use paper tissues	4.03	(1.41)	5	(3–5)	4.17	(1.30)	4	(4–5)	23884	0.394
**Preventive behaviour SI**											
1.	Wash my hands regularly (20 sec.) with soap and water	3.69	(1.07)	4	(3–4)	3.72	(1.03)	4	(3–4)	22463	0.821
2.	Sneeze or cough in my elbow	4.10	(1.07)	4	(4–5)	4.07	(1.16)	4	(4–5)	22552	0.877
3.	Keep 1.5 meters away from other people	3.65	(1.07)	4	(3–4)	3.61	(1.02)	4	(3–4)	23652	0.441
4.	Avoid people who are vulnerable	4.08	(1.00)	4	(4–5)	4.04	(1.09)	4	(4–5)	22859	0.918
5.	Work from home as much as possible	3.59	(1.62)	4	(2–5)	3.48	(1.63)	4	(2–5)	23531	0.511
6.	Avoid crowds	3.88	(1.33)	4	(3–5)	3.76	(1.30)	4	(3–5)	24314	0.193
7.	Use paper tissues	3.97	(1.36)	5	(3–5)	3.86	(1.44)	5	(3–5)	23256	0.658

Note. Mdn = median, n = number of participants, Range: never (1) to always (5). Wilcoxon rank sum tests were performed with continuity correction.

*Significant after (Holm-Bonferroni) correction for multiple testing

### Study 2: Behavioural journalism

In total, 593 of 638 participants consented to participate and were randomly allocated to the behavioural journalism condition (n = 290) or control (n = 303) group. In total, data of 449 participants were analysed (n = 212 intervention, n = 235 control). Dropout among the participants was 25%; this was due to non-response or unreachable e-mail addresses.

Characteristics of participants are presented in Table 1. Socio-demographics were similar for the intervention and control group. There was, however, a small difference between the two groups with regard to sex. Most participants were female, were born in the Netherlands, finished higher education, did not work in healthcare and had someone in their environment with and increased risk of becoming ill from COVID-19.

The manipulation check showed that 37.6% of the participants watched one video, 43.1% two or more videos and 19.3% indicated not to have watched any video. The videos from the male student and the young woman working as specialist in geriatric medicine were most frequently watched (47.6% and 43.1%). Most of the participants reported that the videos were relevant (55.3%) and recognizable (59.3%). Nevertheless, only 10% perceived the stories as helpful.

Tables 2 and 3 show the results of means, standard deviations, and internal consistency of social psychological measures and the results of the regression analyses. We hypothesised that the engagement with behavioural journalism would contribute to an increase in intention and self-efficacy to comply with preventive measures. However, no significant differences were found between the intervention and control group on any of the measures at both post-test and follow-up. Furthermore, there were no differences in compliance with the behavioural measures (see Table 4).

### Study 3: Empathy induction

In total, 578 of 623 participants consented to participate, which were then allocated to the intervention (n = 261) or control (n = 317) group. In total, data of 428 participants were analysed (n = 196 intervention, n = 232 control). Of the participants, 26% dropped out due to non-response or unreachable e-mail addresses.

The characteristics of participants are displayed in Table 1. Both the experimental and the control group were comparable based on socio-demographical characteristics. There was, however, a small difference in age between the two groups, which was corrected for in the analyses. Most participants were Dutch females who finished higher education.

The manipulation check showed that most participants in the intervention group reported to have established feelings of sympathy (72.0%), compassion (71.2%), involvement (56.9%), understanding (76.6%) and warmth (52.7%) when watching the video of the vulnerable woman. Additionally, 82.7% reported to find it important to protect (vulnerable) others and 67.7% reported that they were willing to protect (vulnerable) others by complying to the precautionary measures after watching the video (see S2 Appendix).

When examining the uncorrected tests, participants in the empathy induction group had a higher intention to adhere to the precautionary measures compared to participants in the control group at post-test. Additionally, participants in the empathy induction group perceived an infection with the virus to be more severe and perceived others to be more vulnerable to become infected with COVID-19 compared to participants in the control group. At follow-up, no significant differences were found between participants in the intervention and control group on any of the social psychological determinants or behavioural compliance at follow-up. After adjusting for multiple comparison by means of the Holm-Bonferroni correction, only the effects for perceived susceptibility of others remained.

## Discussion

In the present study we examined strategies to enhance compliance with the COVID-19 behavioural measures.

Based on the three experiments we showed that people exposed to the volitional help sheets were more likely follow-up behavioural measures to avoid other people (limiting visitors at home and avoiding crowds). In addition, people who received the volitional help sheets were more likely to acknowledge the risk of COVID-19 for themselves and for vulnerable others. The empathy induction manipulation enhanced the perception of risk of vulnerable others. Generally, based on conservative analyses adjusted for multiple testing, we were not able to confirm our hypotheses to improve behavioural compliance or its determinants motivation, self-efficacy and intention by means of the three strategies volitional help sheet, behavioural journalism and empathy induction, except for a confirmation of effects on individual behavioural compliance measures by means of the volitional help sheet. However, analyses unadjusted for multiple testing suggested that the volitional help sheet and empathy induction may have potential to improve behavioural compliance: First indications for effects were found for empathy induction on intention to comply with behavioural compliance measures and for the volitional help sheet on self-efficacy, intention and behavioural compliance. Although these effects were small, and short-term, they could be clinically relevant from a public health perspective [27]. Even changing the behaviour of a small percentage of the population could reduce the transmission of COVID-19 and save lives [28]. Furthermore, our findings comply with some other recent findings. For instance, also Pfattheicher et al. [29] showed that highlighting vulnerable persons can encourage physical distancing. Unfortunately, the behavioural journalism strategy did not improve motivation or compliance.

Thus far, a lot of effort has been invested in conveying advice to comply with behavioural measures e.g. [30–32]. Nevertheless, people do not always experience when, where, how and why to comply with the behavioural norms. Especially, when measures change over time. We have shown that behaviour change techniques, derived from health psychology, can be helpful in communicating towards people and supporting people to maintain the behavioural measures.

We did show that two out of three strategies may be effective means to promote (determinants of) compliance to the behavioural measures. We also showed that experimental pilot tests to examine the usefulness of interventions can be piloted rapidly and effectively, which could improve evidence-based advice towards the public, prior to large-scale implementation.

Before discussing further practical implications, we should acknowledge some limitations of our study. Generally, our sample comprised of a convenience sample (recruited by Facebook). Besides, the sample was generally higher educated, the findings may thus not translate to the general public.

Second, we used a post-test control group design with a follow-up at one week. Although, we did not see any demographic differences between the experimental and control group, a pre-post-test control group design with a baseline assessment of the psychosocial determinants and behaviour would have ruled out potential differences, which would provide a stronger case for causation between the intervention and the outcomes.

Another potential limitation may be the lack of validated measures—despite good reliability. Nevertheless, the measures were operationalized in accordance with guidelines [e.g., 33]. Similar measures have been used to also understand behavior and determinants in the context of similar type of infectious diseases [e.g., 4].

Finally, the follow-up at one week does not allow to draw conclusions regarding any longer-term effects on compliance over time. Yet, to minimise participant burden and attrition, we opted for the present design.

In terms of practical implications, we think that both the empathy induction strategy and volitional help sheet could contribute to compliance of preventive COVID-19 measures. A suggestion would be to combine the strategies as the empathy induction might enhance (prosocial) protection motivation: motivation is generally a requirement for the effectiveness of implementation intentions or the VHS [34]. On the other hand, implementation intentions have shown to decrease intention-behaviour gaps, especially for more complex behaviours. Hence, this approach might both motivate and strengthen people to maintain the behavioural measures, by highlighting the importance to protect vulnerable people, and providing the ways to overcome difficult situations. We believe these strategies may be potential means for use during the phase of an outbreak and beyond. Moreover, our study shows that health psychology theory and intervention strategies may provide an important contribution to the more medical or epidemiological science devoted to minimizing the repercussions of the COVID-19 outbreak.

The Behavioural Journalism strategy did not result in favourable changes. Nevertheless, we are hesitant to advice not to use the strategy. Clearly, the manipulation check showed we were able to ensure people could relate to the situations. We did not find a difference between older and younger participants (data not shown), which we checked in order to assess whether the younger aged role models mattered. Nevertheless, the manipulation check also revealed the information gathered was not novel; in addition, one in five people reported that they did not watch any video. Future studies should examine which scenarios would be novel, acceptable and adoptable.

WHO has acknowledged the importance of behavioural sciences in combatting the pandemic [32]. We hope to have demonstrated that health psychology may contribute to preventing the spread of COVID-19. Moreover, we add to the different behavioural monitors being conducted in the Netherlands [31] and elsewhere, by providing means to combat declines in motivation. These strategies can be used by governmental agencies to promote compliance, be it the national government or the local public health centres, when facing outbreaks. The insights at hand remain relevant in times of vaccination as people need to remain compliant with the preventive measures after vaccination.

## Conclusion

In the present study, we showed that health behavioural strategies may have the potential to contribute to prolonged compliance with the behavioural measures. This information could be valuable for both governments, governmental organisations and public health centres in further combating COVID-19, also in times of increased vaccination uptake. Experimental pretesting of behavioural interventions to improve behavioural compliance is useful to ensure more evidence-based communication.

## Supporting information

S1 Appendix Volitional help sheet. (DOCX)

S2 Appendix Manipulation check. (DOCX)
