# Supplementary material for: Improving behavioural compliance with the COVID-19 precautionary measures by means of innovative communication strategies: Social experimental studies
Source: PLoS One. 2022 Jul 28;17(7):e0272001. doi: 10.1371/journal.pone.0272001 (PMC9333445; doi:10.1371/journal.pone.0272001)
Supplement: S1 Appendix — (DOCX) [file pone.0272001.s001.docx]

**Appendix A: Volitional Help Sheet**

| **VHS** | |  | ***n*** | **(%)** |
| --- | --- | --- | --- | --- |
| 1. | When I am in the supermarket and I have less than 1.5 meters to pass someone | |  |  |
|  |  | 1 I will wait until there is enough space to pass | 40 | (38.1) |
|  |  | 2 I will step aside | 46 | (43.8) |
|  |  | 3 I will go during a different time slot next time | 1 | (1.0) |
|  |  | 4 I will have my groceries delivered next time | 3 | (2.9) |
|  |  | 5 I will go to a place where I can keep enough distance to others | 15 | (14.3) |
| 2. | When there is less than 1.5 meters of space when walking | |  |  |
|  |  | 1 I will wait until there is enough space to pass | 2 | (6.1) |
|  |  | 2 I will step aside | 25 | (75.8) |
|  |  | 3 I will go during a different time slot next time | 0 | (0.0) |
|  |  | 4 I will have my groceries delivered next time | 1 | (3.0) |
|  |  | 5 I will go to a place where I can keep enough distance to others | 5 | (15.2) |
| 3. | When someone gets too close | |  |  |
|  |  | 1 I will step aside | 43 | (57.3) |
|  |  | 2 I will ask if that person can keep enough distance | 17 | (22.7) |
|  |  | 3 I will go to a place where I can keep enough distance to others | 15 | (20.0) |
| 4. | When I see / notice that someone needs a hug | |  |  |
|  |  | 1 I will send that person a postcard | 21 | (30.4) |
|  |  | 2 I will (video) call that person | 37 | (53.6) |
|  |  | 3 I will do something nice with that person from distance (a game on the phone) | 11 | (15.9) |
| 5. | When I miss going out for days | |  |  |
|  |  | 1 I will (video) call someone | 4 | (9.1) |
|  |  | 2 I will do something nice with that person from distance (a game on the phone) | 4 | (9.1) |
|  |  | 3 I will do something creative in my own home | 30 | (68.2) |
|  |  | 4 I will remember why I stay at home: to protect myself and others from corona | 6 | (13.6) |
| 6. | When I have a special occasion, such as a birthday or holiday | |  |  |
|  |  | 1 I will send that person/someone a postcard | 29 | (40.8) |
|  |  | 2 I will (video) call that person/someone | 27 | (38.0) |
|  |  | 3 I will do something nice with that person/someone from distance (a game on the phone) | 4 | (5.6) |
|  |  | 4 I will remember why I stay at home: to protect myself and others from corona | 11 | (15.5) |
| 7. | When I feel alone or lonely | |  |  |
|  |  | 1 I will send someone a postcard | 0 | (0.0) |
|  |  | 2 I will (video) call someone | 32 | (52.5) |
|  |  | 3 I will do something nice with someone from distance (a game on the phone) | 4 | (6.6) |
|  |  | 4 I will do something creative in my own home | 20 | (32.8) |
|  |  | 5 I will remember why I stay at home: to protect myself and others from corona | 5 | (8.2) |
| 8. | When I need a hug | |  |  |
|  |  | 1 I will (video) call someone | 19 | (55.9) |
|  |  | 2 I will do something nice with someone from distance (a game on the phone) | 3 | (8.8) |
|  |  | 3 I will remember why I stay at home: to protect myself and others from corona | 12 | (35.3) |
| 9. | When someone is suddenly at the door | |  |  |
|  |  | 1 I will (video) call someone | 0 | (0.0) |
|  |  | 2 I will do something nice with someone from distance (a game on the phone) | 1 | (2.0) |
|  |  | 3 I will say I do not want to not invite that person into my home due to the corona virus | 6 | (11.8) |
|  |  | 4 I will say that it is alright to talk outside, for example in the garden, with enough distance | 44 | (86.3) |
|  |  | 5 I will not open the door | 0 | (0.0) |
| 10. | When I feel lonely and need to see friends or family | |  |  |
|  |  | 1 I will (video) call someone | 23 | (31.1) |
|  |  | 2 I will do something nice with someone from distance (a game on the phone) | 3 | (4.1) |
|  |  | 3 I will remember why I stay at home: to protect myself and others from corona | 10 | (13.5) |
|  |  | 4 I will visit them, but stay outside with enough distance and we will talk through the window | 38 | (51.4) |
| 11. | When I don't have soap in the house | |  |  |
|  |  | 1 I will use desinfectans | 12 | (66.7) |
|  |  | 2 I will put soap in my grocery list | 6 | (33.3) |
| 12. | When I forget to wash my hands | |  |  |
|  |  | 1 I will set an alarm on my phone for every two hours to remind me to wash my hands | 3 | (4.5) |
|  |  | 2 I will put a note on the door to remind me to wash my hands when I come home | 8 | (12.1) |
|  |  | 3 I will put soap on a place where I come regularly | 55 | (83.3) |
| 13. | When family members (grandma, grandpa, mother, father) feel lonely and ask if I come by | |  |  |
|  |  | 1 I will send them a postcard | 6 | (5.9) |
|  |  | 2 I will (video) call them | 33 | (32.4) |
|  |  | 3 I will do something nice with them from distance (a game on the phone) | 1 | (1.0) |
|  |  | 4 I will visit someone, but stay outside with enough distance and we will talk through the window | 62 | (60.8) |
