# Supplementary material for: Improving behavioural compliance with the COVID-19 precautionary measures by means of innovative communication strategies: Social experimental studies
Source: PLoS One. 2022 Jul 28;17(7):e0272001. doi: 10.1371/journal.pone.0272001 (PMC9333445; doi:10.1371/journal.pone.0272001)
Supplement: S2 Appendix — (DOCX) [file pone.0272001.s002.docx]

**Appendix B: Manipulation Check Empathy Induction**

| **What feelings did you have when you saw the interview with Anne-Marie?** | | **Not at all** | | **Little** | | **Neutral** | | **Somewhat** | | **Totally** | | **Missing** |  |
| --- | --- | --- | --- | --- | --- | --- | --- | --- | --- | --- | --- | --- | --- |
|  |  | ***n*** | **(%)** | ***n*** | **(%)** | ***n*** | **(%)** | ***n*** | **(%)** | ***n*** | **(%)** | ***n*** |  |
| 1. | Feelings of sympathy | 8 | (3.1) | 9 | (3.5) | 56 | (21.5) | 80 | (30.8) | 107 | (41.2) | 316 |  |
| 2. | Feelings of compassion | 7 | (2.7) | 24 | (9.2) | 44 | (16.9) | 98 | (37.7) | 87 | (33.5) | 316 |  |
| 3. | Feeling of involvement | 10 | (3.8) | 23 | (8.8) | 79 | (30.4) | 92 | (35.4) | 56 | (21.5) | 316 |  |
| 4. | Feelings of understanding | 9 | (3.5) | 16 | (6.2) | 36 | (13.8) | 79 | (30.4) | 120 | (46.2) | 316 |  |
| 5. | Feelings of warmth | 10 | (3.8) | 24 | (9.2) | 89 | (34.2) | 77 | (29.6) | 60 | (23.1) | 316 |  |
| 6. | We should protect people who are more vulnerable to corona | 18 | (6.9) | 27 | (10.4) | 27 | (10.4) | 95 | (36.5) | 93 | (35.8) | 316 |  |
| 7. | How important do you think it is to prevent vulnerable people from being infected with corona? | 7 | (2.7) | 13 | (5.0) | 25 | (9.6) | 109 | (41.9) | 106 | (40.8) | 316 |  |
| 8. | To what extent are you willing to protect another by following the behavioral measures? | 47 | (18.1) | 29 | (11.2) | 8 | (3.1) | 54 | (20.8) | 122 | (46.9) | 316 |  |
